# SM934 Treated Lupus-Prone NZB×NZW F_1_ Mice by Enhancing Macrophage Interleukin-10 Production and Suppressing Pathogenic T Cell Development

**DOI:** 10.1371/journal.pone.0032424

**Published:** 2012-02-28

**Authors:** Li-Fei Hou, Shi-Jun He, Xin Li, Chun-Ping Wan, Yang Yang, Xiao-Hui Zhang, Pei-Lan He, Yu Zhou, Feng-Hua Zhu, Yi-Fu Yang, Ying Li, Wei Tang, Jian-Ping Zuo

**Affiliations:** 1 Laboratory of Immunopharmacology, State Key Laboratory of Drug Research, Shanghai Institute of Materia Medica, Chinese Academy of Sciences, Shanghai, People's Republic of China; 2 Laboratory of Immunology and Virology, Shanghai University of Traditional Chinese Medicine, Shanghai, People's Republic of China; 3 Department of Synthetic Chemistry, Shanghai Institute of Materia Medica, Chinese Academy of Sciences, Shanghai, People's Republic of China; University of Oklahoma and Oklahoma Medical Research Foundation, United States of America

## Abstract

**Background:**

Artemisinin and its derivatives were reported to possess strong regulatory effects on inflammation and autoimmune diseases. This study was designed to examine the therapeutic effects and underlying mechanisms of SM934, a water-soluble artemisinin analogue, on lupus-prone female NZB×NZW F_1_ mice.

**Methodology/Principal Findings:**

NZB/W F_1_ mice were treated orally with SM934 for 3 or 6 months respectively to investigate the effect on clinical manifestations and immunological correlates. To further explore the mechanisms of SM934, ovalbumin (OVA)-immunized or interferon (IFN)-γ-elicited C57BL/6 mice were used. *In vivo*, treatment with SM934 for 3 or 6 months significantly delayed the progression of glomerulonephritis and increased the survival rate of NZB/W F_1_ mice. Clinical improvement was accompanied with decreased Th1-related anti-double-strand DNA (dsDNA) IgG2a and IgG3 Abs, serum interleukin (IL)-17, and increased Th2-related anti-dsDNA IgG1 Ab, serum IL-10 and IL-4. SM934 treatment also suppressed the accumulation of effector/memory T cells, induced the apoptosis of CD4^+^ T cells, while enhancing the development of regulatory T cells in NZB/W F_1_ mice. In addition, SM934 treatment promoted the IL-10 production of macrophages from NZB/W F_1_ mice, OVA-immunized C57BL/6 mice and IFN-γ-elicited C57BL/6 mice. *In vitro*, SM934 enhanced IL-10 production from primary macrophages stimulated with IFN-γ.

**Conclusions/Significance:**

The results of this study demonstrated that artemisinin analogue SM934 had therapeutic effects on lupus-prone female NZB/W F_1_ mice by inhibiting the pathogenic helper T cell development and enhancing anti-inflammatory cytokine IL-10 production.

## Introduction

Systemic lupus erythematosus (SLE) is a chronic autoimmune disease characterized by abnormal accumulation of autoreactive T lymphocytes and production of autoantibody against self-antigen, which result in the development of immune complex-mediated glomerulonephritis and renal failure [1], [2]. Female NZB/W F_1_ mice resemble human lupus closely. This strain of mice spontaneously develops severe autoimmune disease, especially the fatal immune complex-mediated glomerulonephritis, around 5 to 7 months of age, and approximately 50% die by 8 months and 90% die by 12 months of age [3], [4]. It's well documented that activated/memory helper T cells (CD4^+^CD44^+^CD62L^−^ effector T cells, Teff), especially IFN-γ-producing Th1 subset, are responsible for inciting human and murine lupus, in part through increased production of highly nephritogenic Th1-related Ig2a and IgG3 autoantibodies [5]–[8]. With the development of pathogenic helper T cells, CD4^+^Foxp3^+^ regulatory T cells (Treg) failed to maintain a competitive pool size in the peripheral lymphoid organs, finally resulting in a progressive imbalance of Treg and Teff [9]–[11].

The recently defined IL-23/IL-17 axis, as well as the IL-12/IFN-γ axis, is emerging to be critical in many autoimmune diseases [12], [13]. IL-23 can directly elicit IL-17 family cytokines from memory helper T cells and other innate immune cells [12], [13], especially the γδ T cells [14], [15]. In addition to recruiting neutrophils, recent evidences also strongly suggest that IL-23/IL-17 axis can exert its function by orchestrating adaptive Th1 responses in inflammatory bowel disease [16], [17], delayed type hypersensitivity [17], [18], and intracellular bacterial infection [19]. Currently, there are compelling evidences for a pathogenic role of IL-17 in human lupus patients and lupus-prone female MRL*/lpr* mice [20], [21]. However, the definite IL-17-producing cells and pathogenic role of IL-17 in lupus are still uncertain.

Disease severity in human and murine lupus is also tightly correlated to IL-10 [22]. It was reported that patients with lupus produced large amount of IL-10 [23], [24]. Administration of anti-IL-10 mAb to human patients with active lupus [25] or NZB/W F_1_ mice [26] led to the amelioration of disease activity. However, there were also some reports supporting a protective role of IL-10 in SLE. For example, in MRL*/lpr* mice, deficiency of IL-10 resulted in exacerbated disease, demonstrated by enhanced Th1 cell development and increased mortality [27]; Over-expression of IL-10 in lupus-prone NZM2410 mice could ameliorate lupus diseases [28]. Routinely, IL-10 is deemed a regulatory cytokine. IL-10 could inhibit IFN-γ production and proliferation of CD4^+^ T cells through its direct inhibitory effects on T cells or indirect inhibitory effects on antigen-presenting cells [29]. Many cells produce IL-10, especially the myeloid cells, and IL-10 released from myeloid cells can maintain Foxp3 expression and suppressive function of Treg cells in a paracrine manner [30]. Thus, the role of IL-10 remains controversial in SLE.

Multifactorial pathogenesis resulted in scarcity of therapeutic approaches for SLE. Artemisinin derivatives possess strong anti-inflammatory and immunosuppressive functions, and have shown significant therapeutic effects on SLE clinically and experimentally [31]–[38]. SM934, β-aminoarteether maleate, was synthesized from β-hydroxyarteether at Shanghai Institute of Materia Medica. Chemically, as the derivative of artemisinin, SM934 also contains the unique peroxide bridge, but has higher bioavailability. Recently, we demonstrated that *in vitro* SM934 could suppress the Th1 and Th17 polarization, but exerted no influence on Treg differentiation; *In vivo*, SM934 could significantly ameliorate lupus diseases in MRL*/lpr* mice through inhibiting the both of Th1 and Th17 responses, and elevating Treg percentage [38]. In MRL*/lpr* mice, the *lpr* (lymphoproliferative) mutation of the *fas* gene impairs activation-induced cell death (AICD), which results in the abnormal accumulation of autoreactive T, B and double negative T lymphocytes (CD3^+^CD4^−^CD8^−^B220^+^ cells) [39]. However, in NZB/W F_1_ mice, the *fas* gene is intact, which makes the pathogenesis of NZB/W F_1_ mice largely different from that of MRL*/lpr* mice. In this study, we explored the therapeutic effects and underlying mechanisms of SM934 on NZB/W F_1_ mice and demonstrated that SM934 could exert comprehensive therapeutic effects on NZB/W F_1_ mice both in short-term and long-term treatment. SM934 treatment could significantly increase Treg percentage and suppress the Th1 and Th17 responses in NZB/W F_1_ mice. Although our previous report suggested that SM934 could induce activated CD4^+^ T cells into apoptosis *in vitro* [34], it's hardly to present such effects of SM934 *in vivo*, for that, usually apoptotic cells will be phagocytized and cleared quickly. Herein, through flow cytometric staining surface B220 and western blot analysis of Bcl-2 protein expression in the CD4^+^ T cells, we firstly demonstrated that SM934 treatment could prompt the apoptosis of activated CD4^+^ T cells *in vivo*. Furthermore, the therapeutic effects of SM934 on NZB/W F_1_ mice were tightly linked to enhancing IL-10 production from macrophages, which was absent in MRL*/lpr* mice.

Collectively, the results of this study demonstrated the therapeutic effects of artemisinin derivative SM934 on female NZB/W F_1_ mice with established nephritis. SM934 treatment could correct pathogenic helper T cell commitment and enhance IL-10 production that might be beneficial for future lupus treatment.

## Results

### Therapeutic effects of SM934 on female NZB/W F_1_ mice with three months oral administration

**Figure 1:**
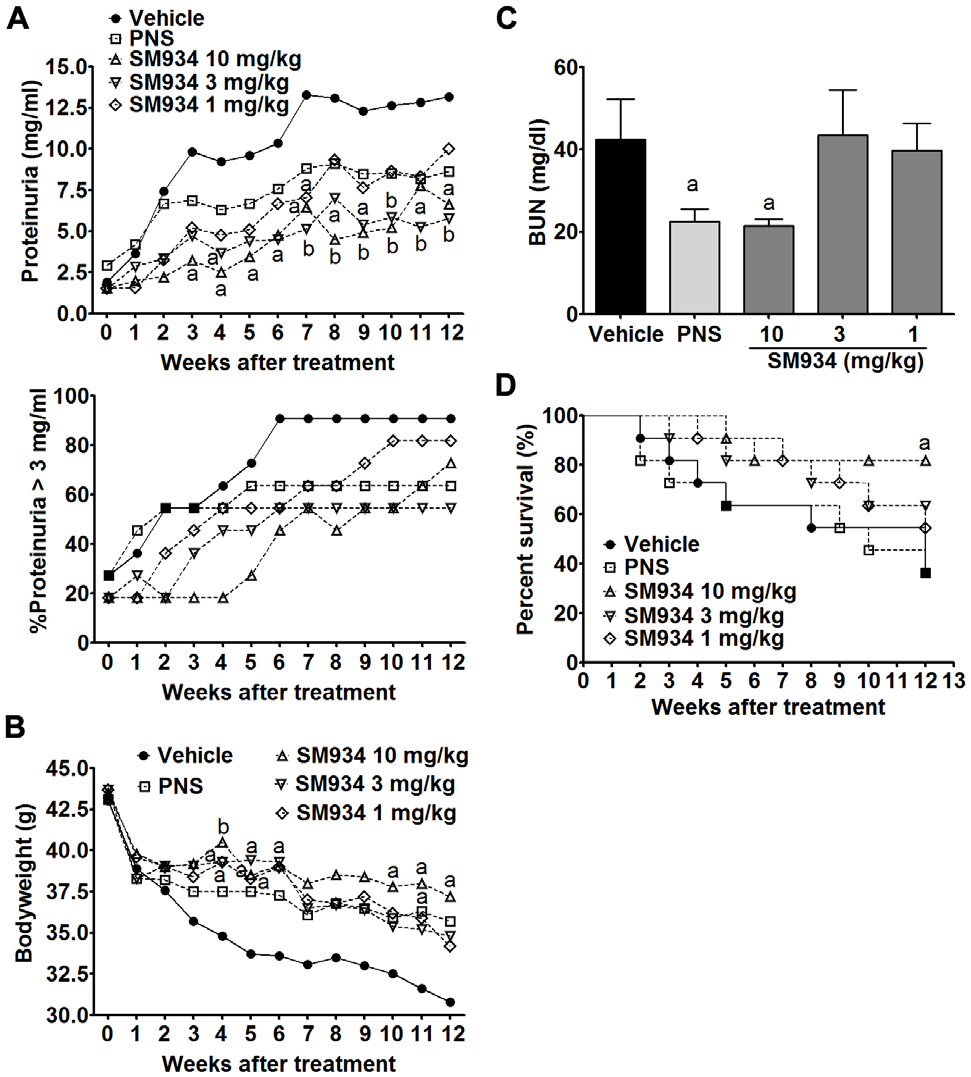
SM934 treatment for 3 months improved lupus syndroms. Six and half months old female NZB/W F_1_ mice were orally treated with vehicle (saline), SM934 (10, 3, and 1 mg/kg, respectively), and PNS (2 mg/kg) for 3 months (n = 11 per group). (A) Up: levels of proteinuria; Bottom: frequencies of mice with severe proteinuria (urinary protein ≥3 mg/ml). (B) Body weight. (C) Serum levels of blood urea nitrogen (BUN) immediately measured at the end of experiment. (D) Cumulative survival rate during 3 months of treatment. a = *P*<0.05; b = *P*<0.01 versus vehicle.

**Figure 2:**
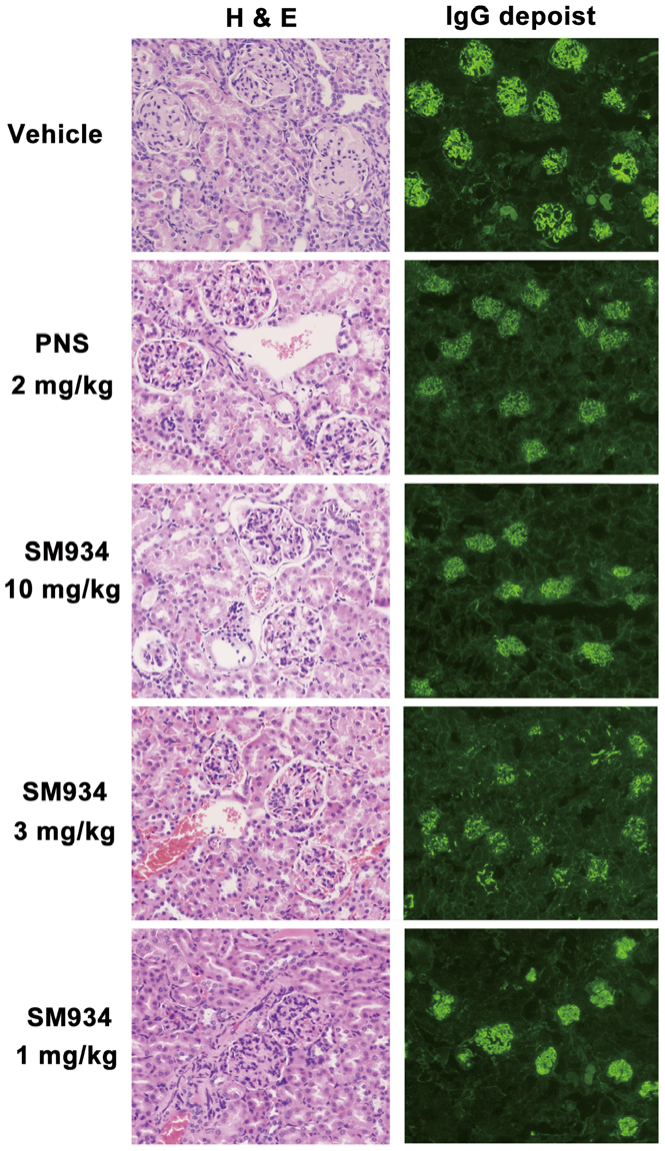
SM934 treatment for 3 months improved kidney injuries and IgG depositions. Six and half months old female NZB/W F_1_ mice were orally treated with vehicle (saline), SM934 (10, 3, and 1 mg/kg, respectively), and PNS (2 mg/kg) for 3 months (n = 11 per group). Left, representative kidney sections stained with H&E (Original magnification×200); Right, representative immunofluorescence staining of IgG deposit in the kidney sections (Original magnification×100).

Female NZB/W F_1_ mice develop kidney inflammation gradually from about 5 months old on. Here, we used 6.5 months old female NZB/W F_1_ mice with ongoing kidney injury, which was demonstrated by about 26% of mice with severe proteinuria scored ≥3+. As shown in Figure 1A, with aging, vehicle treated mice progressively developed severe proteinuria, manifested by absolute urinary protein concentration (Up-panel) and percentage of severe proteinuria scored ≥3+ (Bottom-panel), and peaked nearly at the age of 8 months. SM934 treatment, both 10 and 3 mg/kg, could dramatically inhibit the progression and aggravation of proteinuria, as quickly as 2 (for 10 mg/kg) to 4 weeks (for 3 mg/kg) after treatment. Meanwhile, SM934 at the dose of 1 mg/kg also presented mild therapeutic effects. In addition, all three doses of SM934 could maintain the bodyweight of lupus-suffered mice during treatment, in which 10 and 3 mg/kg showed statistical significance at indicated time point (Figure 1B). At the end of 3 months of treatment, mice were sacrificed and examined for blood urea nitrogen (BUN). Results showed that SM934 (10 mg/kg) and prednisolone (PNS) treatment significantly decreased the BUN levels (Figure 1C). Accumulated survival rate showed that SM934 treatment, especially at the dose of 10 mg/kg, could significantly decrease the death rate (p<0.05, Figure 1D). Kidneys were examined to evaluate renal pathology and IgG deposition. Histological sections from vehicle treated mice exhibited severe renal damage, characterized by glomerular sclerosis, hyalinosis, increased mesangial matrix, diffused perivascular and interstitial mononuclear cell infiltration, tubular atrophy, and accumulation of proteinaceous casts in the tubules. Mice treated with SM934 (10 and 3 mg/kg) presented significantly less severe renal damage that showed marked amelioration in glomerular, perivascular injuries, and interstitial inflammatory cell infiltration and lesion (representative histopathological pictures in Figure 2, Left panel and detailed individual histological score was listed in Table S1).

Renal cryostat sections were stained for detecting IgG deposition. The pronounced mesangial IgG deposits were shown in kidneys from vehicle treated mice. In contrast, mice treated with SM934 (10 and 3 mg/kg) showed remarkably diminished IgG deposition (representative immunofluorescence pictures in Figure 2, Right panel and detailed individual score was listed in Table S1).

### Effects of SM934 treatment on the sera levels of autoantibodies and cytokines in female NZB/W F_1_ mice

**Figure 3:**
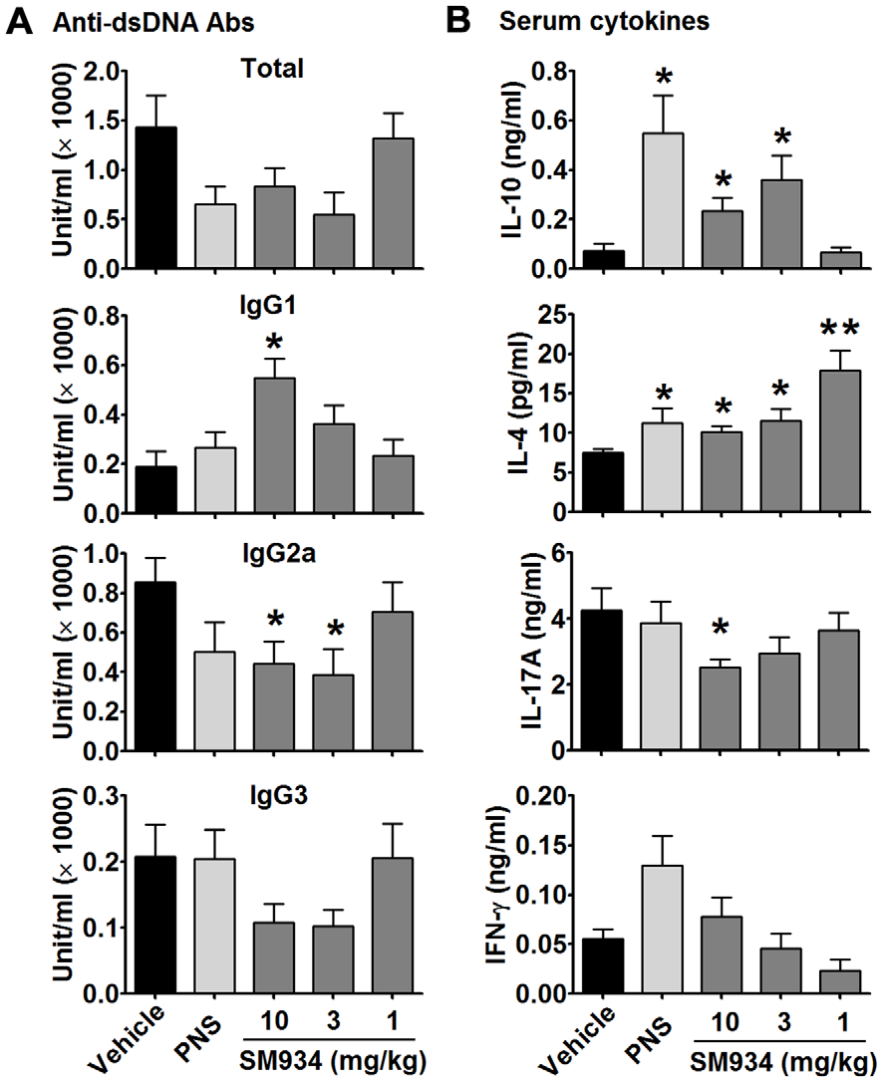
SM934 treatment regulated anti-dsDNA autoantibodies and cytokines in sera. At the termination of 3 months treatment, sera of female NZB/W F_1_ mice were collected and examined for anti-dsDNA autoantibodies and cytokines. (A) Serum levels of anti-dsDNA total IgG, IgG1, IgG2a and IgG3 antibodies. Sera of 10 months old female NZB/W F_1_ mice were used as the standard control. (B) Serum levels of IL-10, IL-4, IL-17A and IFN-γ. Shown were mean ± SEM. * = *P*<0.05; ** = *P*<0.01 versus vehicle.

Anti-dsDNA IgG autoantibodies, especially Th1-related IgG2a and IgG3 isotypes, are hallmarks of SLE and play important pathogenic roles in lupus nephritis [5]–[8], [40]. However, Th2-related IgG1 isotype might play a protective role in murine and human lupus through preferentially stimulating inhibitory FcγR [40], [41]. The serum levels of anti-dsDNA antibodies were examined and shown in Figure 3A. Three months SM934 treatment, at the doses of 10 and 3 mg/kg, largely decreased total anti-dsDNA IgG Ab (p = 0.13 and p = 0.05, respectively), IgG2a isotype (both p<0.05) and IgG3 isotype (p = 0.12 and p = 0.09, respectively), but significantly increased the level of IgG1 isotype (p<0.01 and p = 0.09, respectively).

The serum levels of cytokines were shown in Figure 3B. SM934 treatment, at the doses of 10 and 3 mg/kg, significantly increased serum levels of IL-10 and IL-4 (p<0.05, respectively). At the dose of 10 mg/kg, SM934 significantly decreased the serum level of IL-17A (P<0.05). However, SM934 treatment exerted no influence on serum transforming growth factor-β (data not shown).

### SM934 treatment prompted the apoptosis of CD4^+^ T cells

**Figure 4:**
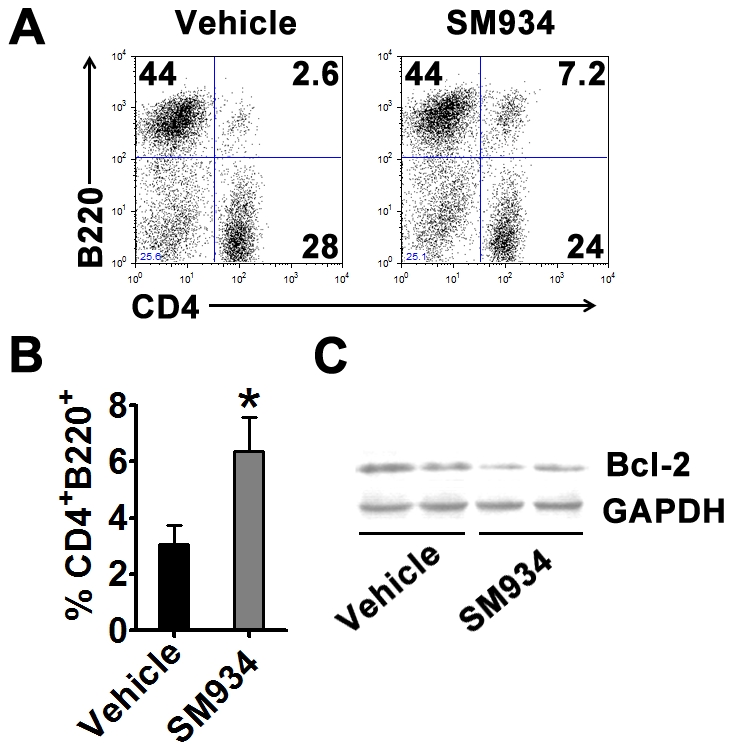
SM934 treatment induced the apoptosis of CD4^+^ T cells. At the termination of 3 months treatment, female NZB/W F_1_ mice in vehicle and SM934 (10 mg/kg) treated groups were randomly and averagely divided into 3 sub-groups. Spleens of each sub-group were pooled together. (A) Representative staining of surface CD4 and B220 in splenocytes. (B) Statistical results of percentage of CD4^+^B220^+^ population in splenocytes. (C) CD4^+^ T cells were purified through magnetic negative selection and examined for Bcl-2 expression through western blot analysis. One blot was the mix of two equal splenic samples from treated groups. Shown are mean ± SD, n = 3 in each treated group. * = *P*<0.05 versus vehicle.

After 3 months of treatment, splenocytes were obtained to examine the immunologic correlates. Flow cytometry analysis showed that SM934 treatment did not influence the percentage of major cellular population in spleens including CD4^+^ T cells, CD8^+^ T cells, B cells, dendritic cells, and monocytes (Table S2). For that our previous study showed that *in vitro* SM934 could exclusively induce activated, not naïve, CD4^+^ T cell into apoptosis [34], we then examined the B220 expression on the surface of CD4^+^ T cells, which was reported to be the apoptotic marker for activated CD4^+^ T cells [42], [43]. Dramatically, SM934 treatment significantly enhanced the accumulation of CD4^+^B220^+^ population in the spleen (Figure 4A and 4B). Furthermore, western blot analysis showed that SM934 treatment decreased the Bcl-2 protein level in purified CD4^+^ T cells (Figure 4C).

### SM934 treatment induced the accumulation of regulatory T cells

**Figure 5:**
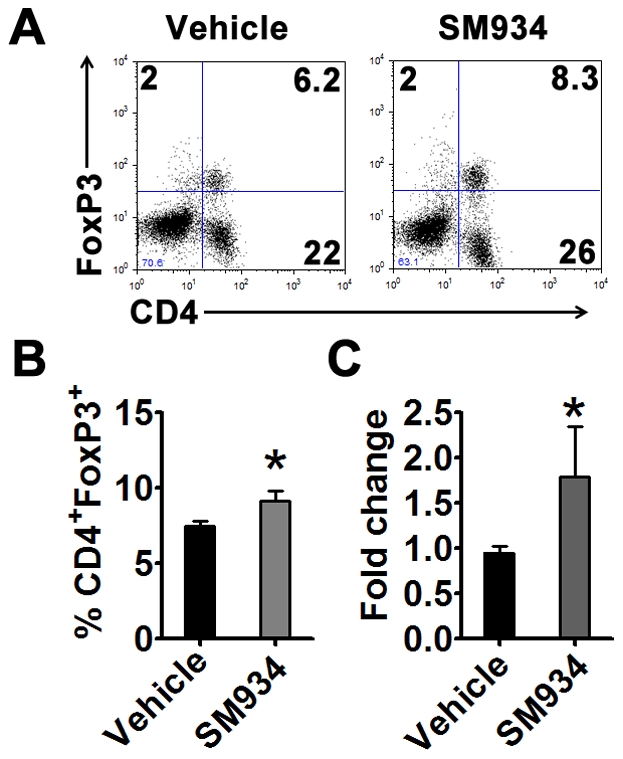
SM934 treatment induced the accumulation of Treg cells. At the termination of 3 months treatment, female NZB/W F_1_ mice in vehicle and SM934 (10 mg/kg) treated groups were randomly and averagely divided into 3 sub-groups. Spleens of each sub-group were pooled together. (A) Surface CD4 and intracellular FoxP3 expressions in splenocytes. (B) Statistical results of percentage of CD4^+^FoxP3^+^ population in splenocytes. (C) Real-time PCR analysis of FoxP3 mRNA expression in purified CD4^+^ T cells. Shown are mean ± SD, n = 3 in each treated group. * = *P*<0.05 versus vehicle.

Our previous study showed that, *in vitro*, SM934 will not influence the differentiation of Treg cells, but could favor the accumulation of Treg cell in the spleens of MRL/lpr mice *in vivo*. We then examined the FoxP3 expression in the splenocytes. Results showed that SM934 treatment significantly enhanced the accumulation of CD4^+^FoxP3^+^ Treg cells in the spleens, regarding both intracellular FoxP3 protein (Figure 5A and 5B) and FoxP3 mRNA levels (Figure 5C) in CD4^+^ T cells.

### Effects of SM934 treatment on the balance of pro-inflammatory and anti-inflammatory cytokines

**Figure 6:**
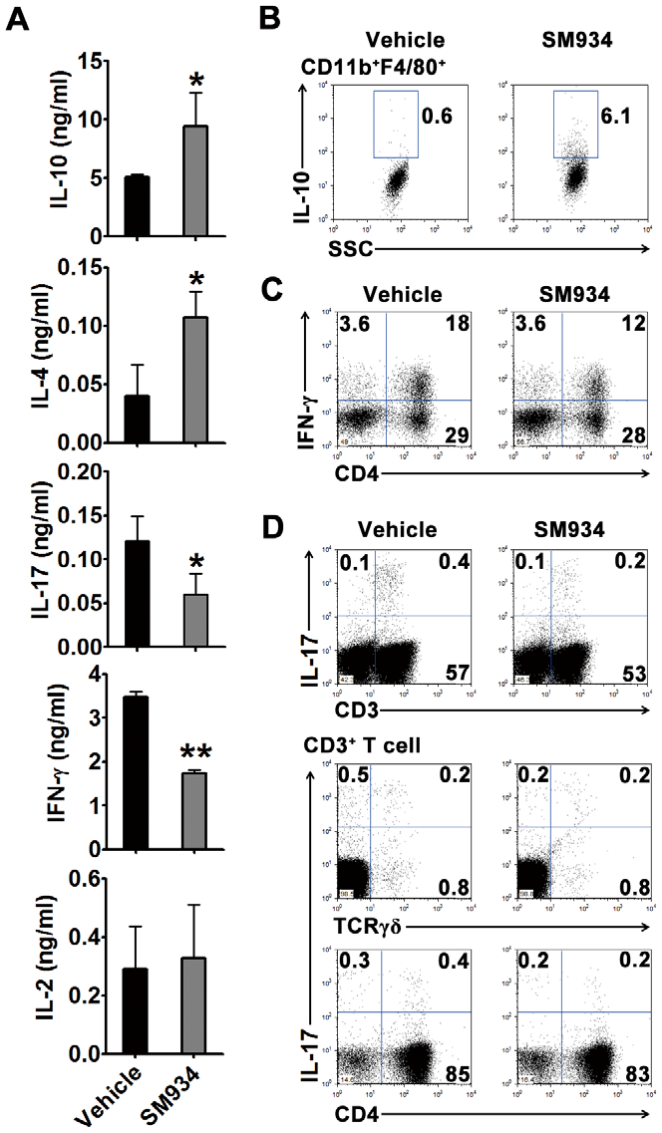
SM934 treatment suppressed Th1 and Th17 cell development and enhanced IL-10 production from macrophages. At the termination of 3 months treatment, female NZB/W F_1_ mice in vehicle and SM934 (10 mg/kg) treated groups were randomly and averagely divided into 3 sub-groups. Spleens of each sub-group were pooled together and *ex vivo* stimulated. (A) Cytokine productions in splenocytes stimulated with anti-CD3 mAb for 24 hours. Shown are mean ± SD, n = 3 in each treated group. (B) Flow cytometric analysis showing intracellular IL-10 expression in CD11b^+^F4/80^+^ macrophages. (C) Flow cytometric analysis of CD3^+^IFN-γ^+^ cells in splenocytes. (D) UP-panel, flow cytometric analysis of CD3^+^IL-17^+^ cells in splenocytes; Bottom-panel, flow cytometric analysis of intracellular IL-17 associated with surface CD4 and TCRγδ expressions. For all flow cytometry plots, shown are representative one of 3 samples with similar pattern in each treated group. * = *P*<0.05, ** = *P*<0.01 versus vehicle.

To confirm the influence of SM934 treatment on the development of pathogenic T cells, splenocytes were *ex vivo* stimulated with anti-CD3 mAb. Results showed that splenocytes of SM934 treated mice produced higher IL-10 and IL-4, and less IL-17 and IFN-γ, compared with vehicle treated ones (Figure 6A). To clarify the major cellular source of IL-10, splenocytes were directly stimulated with PMA and ionomycin and examined by intracellular staining and flow cytometric analysis. The result was shown in Figure 6B, SM934 treatment significantly enhanced CD11b^+^F4/80^+^ macrophages, but not CD11b^+^CD11c^+^, CD4^+^, CD8^+^, B220^+^ cells (data not shown), to produce IL-10. Furthermore, flow cytometric analysis showed that SM934 treatment reduced the accumulation of IFN-γ-producing CD4^+^ T cells (Figure 6C) and IL-17-producing CD3^+^ T cells in the spleens (Figure 6D, Up-panel). Through co-staining of different surface markers, we identified that SM934 treatment only decreased the percentage of IL-17^+^ population in CD3^+^γδ^−^ T cells, but not in CD3^+^γδ^+^ T cells (Figure 6D, Bottom-panel).

### SM934 treatment induced IL-10 production from macrophage in OVA-immunized mice

**Figure 7:**
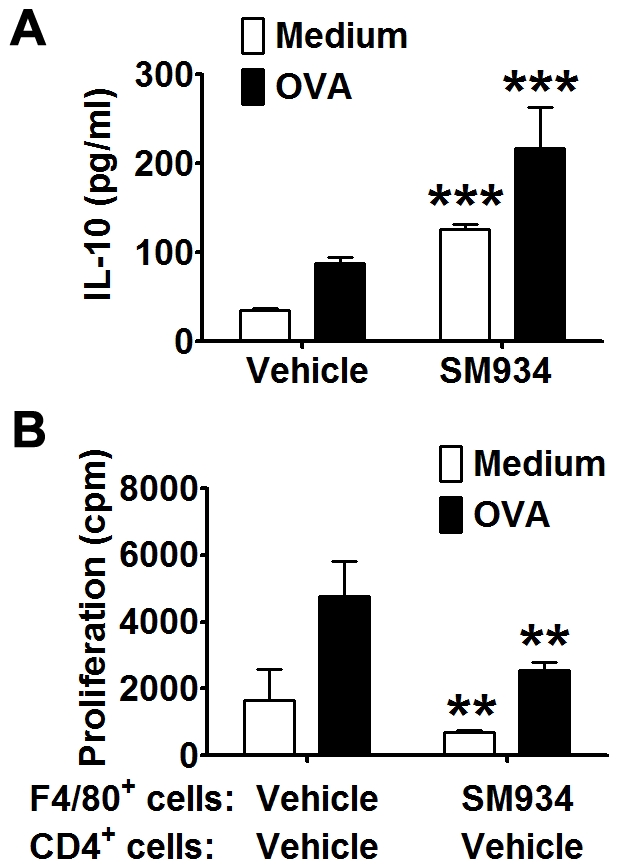
SM934 treatment induced IL-10 production of macrophages in OVA-immunized mice. Naïve female C57BL/6 mice were immunized with OVA/CFA and treated with vehicle or SM934 (10 mg/kg) for consecutive 7 days (9 mice per treated group). At the termination of treatment, both groups were randomly and averagely divided into 3 sub-groups. Spleens of each sub-group were pooled together. (A) IL-10 production in splenocytes cultured with OVA recall for 24 hours. (B) OVA-induced proliferation in CD4^+^ T cells from vehicle treated mice co-cultured with macrophages from vehicle or SM934 treated mice for 72 hours. Bars showed the mean ± SD. ** = *P*<0.01, *** = *P*<0.001 versus corresponding medium or vehicle control with similar stimulatory condition.

To further confirm the effect of SM934 that induced higher production of IL-10 in macrophages, OVA/CFA-immunized female C57BL/6 mice were treated with SM934 (10 mg/kg) or vehicle for 7 days. Then, the splenocytes were obtained and stimulated with or without OVA recall. Compared with splenocytes from vehicle treated mice, splenocytes from SM934 treated mice produced more IL-10 (Figure 7A). In addition, purified F4/80^+^ splenic macrophages from SM934 treated mice showed the suppressive effect on the proliferation of co-cultured CD4^+^ T cells purified from vehicle treated mice under OVA-recall stimulation (Figure 7B).

### SM934 induced IL-10 production from macrophage elicited by IFN-γ *in vitro* and *in vivo*

**Figure 8:**
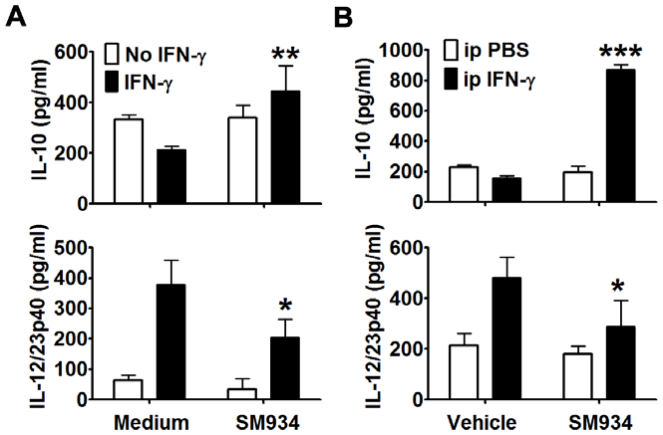
SM934 induced IL-10 production from IFN-γ-challenged macrophages *in vitro* and *ex vivo*. (A) Peritoneal macrophages were isolated from naïve C57BL/6 mice and then were *in vitro* stimulated with mIFN-γ in the presence or absence of SM934 for 24 hours. IL-10 (UP-panel) and IL-12/23p40 (Bottom-panel) productions in the supernatants were examined by ELISA. (B) C57BL/6 mice were *i.p.* elicited with mIFN-γ and orally administrated with vehicle or SM934 (10 mg/kg) for 3 days. Then, peritoneal macrophages were cultured with medium for 24 hours and supernatant IL-10 (UP-panel) and IL-12/23p40 (Bottom-panel) were examined by ELISA. Bars showed the mean ± SD of 3 independent experiments. * = *P*<0.05, ** = *P*<0.01, *** = *P*<0.001 versus corresponding medium or vehicle control with similar stimulatory condition.

Enriched primary peritoneal macrophages obtained from naïve female C57BL/6 mice were *in vitro* stimulated with or without IFN-γ to further determine the effect of SM934. The results showed that SM934 (10 µM) could directly enhance IL-10 production and suppress IL-12/23p40 production in primary peritoneal macrophages with IFN-γ stimulation (Figure 8A).

To further confirm the IL-10-inducing ability of SM934 on IFN-γ-stimulated macrophages, we *i.p.* challenged the mice with IFN-γ and treated them with SM934 for 3 days. As shown in Figure 8B, e*x vivo*, SM934 treatment also significantly enhanced IL-10 production and suppressed IL-12/23p40 production from peritoneal macrophages.

### Long-term treatment of SM934 gained more valuable therapeutic effects on female NZB/W F_1_ mice

**Figure 9:**
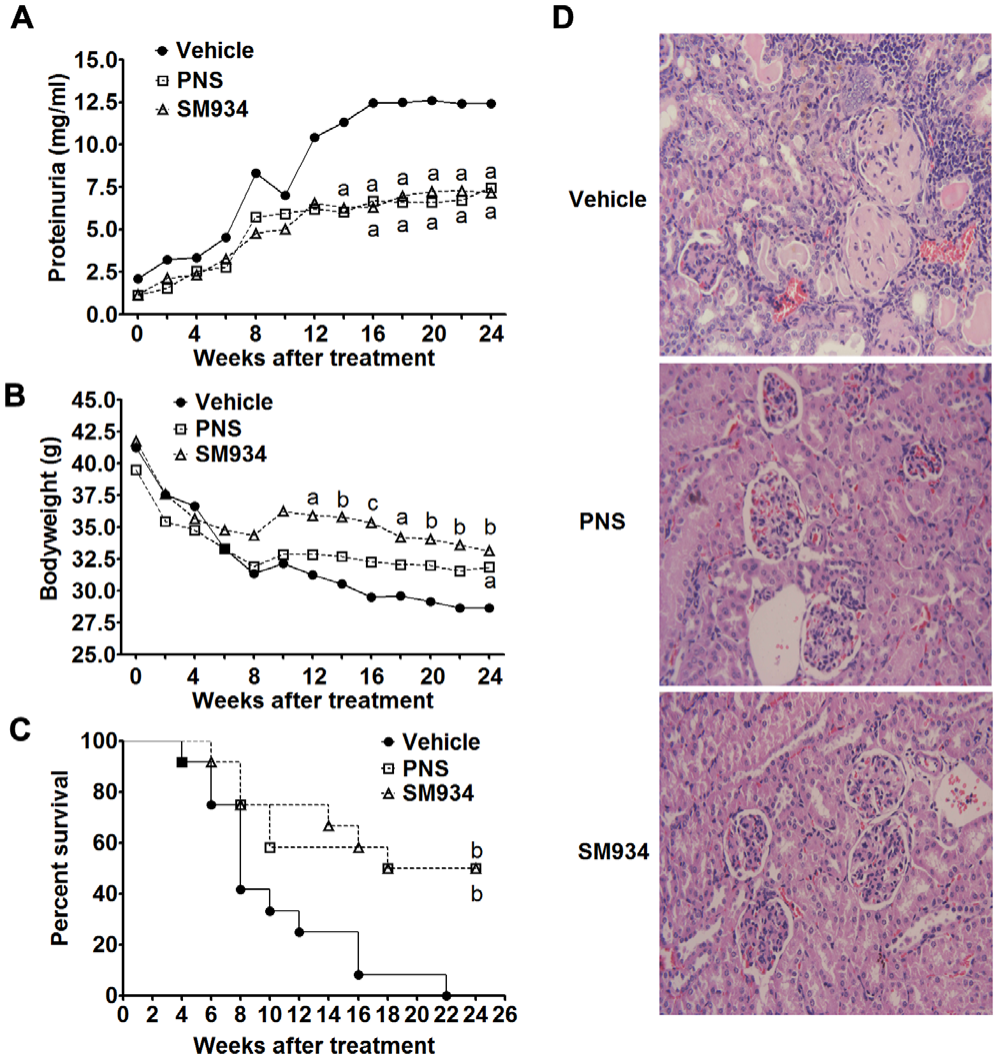
SM934 treatment for 6 months significantly prevented fatal nephritis and prolonged the lifespan. Six and half months old female NZB/W F_1_ mice were orally treated with vehicle, SM934 (10 mg/kg), and PNS (2 mg/kg) for 6 months (n = 12 per group). (A and B), The levels of proteinuria (A) and bodyweight (B) were measured every two weeks. (C) Cumulative survival rate during 6 months of treatment. (D) Histology of representative kidney sections stained with H&E (Original magnification×200). In A, B and C, a = *P*<0.05; b = *P*<0.01; c = *P*<0.001 versus vehicle.

To examine the long-term therapeutic effects of SM934, 6.5 months old female NZB/W F_1_ mice were treated with SM934 for 6 months at the dose of 10 mg/kg. Results showed that, with aging, vehicle treated mice developed severe proteinuria accompanied with progressive bodyweight loss. Compared with vehicle group, SM934 treatment significantly suppressed the progressive elevation of proteinuria level (Figure 9A) and ameliorated the loss of bodyweight (Figure 9B) during 6 months treatment period. Accumulated survival rate showed that SM934 treatment significantly prolonged the lifespan (Figure 9C). After 5.5 months treatment (at the age of 12 months), all mice in vehicle group died, while nearly 50% in SM934 treated group was still alive even after 6 months treatment. At the termination of this 6 months treatment, all remained mice were humanely sacrificed, and kidneys were removed for histological evaluation. As shown in Figure 9D, SM934 treatment significantly alleviated the kidney lesions, especially for decreased glomerular mesangial proliferation, basement membrane proliferation, glomerular sclerosis, tubular dilatation, and perivascular injuries, but exerted minor influence on interstitial inflammatory (detailed histological score was listed in Table S3).

## Discussion

In this study, we explored the therapeutic effects and mechanisms of SM934 on autoimmune syndrome in lupus-prone female NZB/W F_1_ mice. Results of this study showed that SM934 treatment could significantly reverse the imbalance of Th1/Th2-related anti-dsDNA autoantibodies level, correct the abnormal development of helper T cells, reduce the immune complex deposition, ameliorate the lethal renal injury, and thus prolong the lifespan of female NZB/W F_1_ mice. Remarkably, the therapeutic effects of SM934 were also tightly linked to the enhanced production of IL-10 from macrophages.

In murine lupus, IgG2a autoantibody selectively binds activatory FcγRIV, while IgG1 isotype preferentially engages inhibitory FcγRIIb [40], [41]. IgG3 autoantibody is the most potent isotype to activate complement system in the kidney. In the current study, SM934 could significantly suppress the serum levels of IgG2a and IgG3 anti-dsDNA autoantibodies, while increase the IgG1 anti-dsDNA subclass, which might largely contribute to the disease alleviation. However, 10 mg/kg SM934 was less effective to suppress the total anti-dsDNA IgG antibody level than 3 mg/kg SM934 did, which might be resulted from the elevated IgG1 subclass. Despite the less potency in suppressing serum total anti-dsDNA autoantibody, 10 mg/kg SM934 exerted similar inhibitory effects with 3 mg/kg SM934 on IgG deposit in the kidneys. Such inconsistency between serum total anti-dsDNA autoantibody level and IgG deposit in the kidney seems to be acceptable, for that, recently, Hughes et al also reported this phenomenon in progesterone treated NZB/W F_1_ mice [44].

IL-17 was found to be an important pathogenic factor in murine and human lupus. In the current report, we also found that there was a high concentration of IL-17 in the serum, and that the therapeutic effects of SM934 were tightly linked with its inhibiting serum IL-17 in NZB/W F_1_ mice. However, the definite cellular source of IL-17 was still elusive in NZB/W F_1_ mice, considering that both Th17 cells and innate-like IL-17-producing γδ T cells were reported to be major IL-17 producers in many autoimmune diseases [14], [15]. In this study, we found that most of IL-17^+^ cells in the spleen were CD3^+^TCRγδ^−^ T cells, which suggested that Th17, rather than γδ T cells were major IL-17 producers at the later stage of disease. SM934 could significantly suppress the development of Th17 cells and decrease serum IL-17 level, but did not exert influence on IL-17^+^γδ T cells. Thus, this study supported that Th17 cells might play an important pathogenic role in lupus diseases.

This pharmacological study also argued the role of so-called pathogenic role of IL-10 in the pathogenesis of lupus. Some published work reported that, in lupus, pathogenic T cells were comprehensive activated and less sensitive to the suppression of Treg or IL-10 [45]–[47]. Recently, Yuan et al demonstrated that the response of monocytes from lupus patients were less sensitive to the suppression of IL-10 [48]. Here, although we did not compare the sensitivity of Teff cells to Treg and IL-10 between vehicle and SM934 treated groups, we still presumed that simultaneously regulating balance of effector T, naïve T, and regulatory T cells and increasing IL-10 production should largely contribute to disease remission. In addition, from the current study and other reports, we propose that IL-10 still work as an immunosuppressive factor, whereas the final readout of the suppressive effects of IL-10 will be determined by the pathogenic status of other responsive cells.

*In vivo*, SM934 treatment could induce the accumulation of Treg cell in peripheral. However, this inducible effect was not likable to be direct. Our previous work showed that, *in vitro*, SM934 could suppress Th1 and Th17 cell differentiation, but exerted no suppressive influence on Treg cell differentiation. Certainly, *in vitro*, SM934 will not induce FoxP3 expression in CD4^+^ T cells. *In vitro*, we also compared the effects of SM934 on the proliferation of CD4^+^CD25^−^ and CD4^+^CD25^+^ T cells isolated from naïve C57BL/6 mice, and finally found that SM934 suppressed the proliferation of both similarly (unpublished observations). Thus, we suggested that, *in vivo*, SM934 treatment induced the apoptosis of activated effector T cells, rather than preferably inducing Treg accumulation, then accelerated the replenishment of CD4^+^ T cell pool consisting of more naïve population.

Collectively, this study demonstrated that artemisinin derivative SM934 exerted valuable therapeutic effects on lupus-prone female NZB/W F_1_ mice and possessed the great potential for future lupus treatment. The therapeutic mechanism of SM934 might be attributed to its regulating development of helper T cell subsets and enhancing IL-10 production.

## Materials and Methods

### Animal experiments

Female NZB/W F_1_ mice were purchased from the Jackson Laboratory. Female C57BL/6 mice were obtained from Shanghai Laboratory Animal Center of the Chinese Academy of Sciences, and were used at 7 to 10 weeks of age. All mice were housed under specific pathogen-free conditions. Experiments were carried out according to the National Institute of Healthy Guide for Care and Use of Laboratory Animals and were approved by the Institutional Animal Care and Use Committee at the Shanghai Institute of Materia Medica, Chinese Academy of Sciences (IACUC protocol # 2010-10-ZJP-01).

### NZB/W F_1_ mice

Six and half months old female NZB/W F_1_ mice were orally treated with SM934 for 3 months or 6 months respectively. At the initiation of treatment, mice were randomly divided according to bodyweight and proteinuria level and were orally treated for consecutive 6 days every week. In 3 months treatment, mice were divided into 5 groups (n = 11 per group): vehicle control group (saline), positive control group (prednisolone, PNS, 2 mg/kg/day), and SM934 treated groups (10, 3, and 1 mg/kg/day respectively), and the bodyweight and proteinuria level were monitored weekly. In 6 months treatment, mice were divided into 3 groups (n = 12 per group): vehicle control group (saline), positive control group (prednisolone, PNS, 2 mg/kg/day), and SM934 treated group (10 mg/kg/day), and the bodyweight and proteinuria level were monitored biweekly. During the treatment, individuals were euthanized, then sera and kidneys were collected, if their urine protein concentration >10 mg/ml for consecutive two weeks accompanied by weight loss >20%. In this case, the last known values for urinary protein and bodyweight were carried forward. For investigations of effects of SM934 treatment on immunological correlates, 3 months treatment experiments were carried out.

### OVA-immunized C57BL/6 mice

Naïve female C57BL/6 mice were immunized with emulsive OVA/CFA according to our previous method [34], then were treated with saline or SM934 (10 mg/kg/day) for 7 days.

### IFN-γ elicited C57BL/6 mice

To investigate the effects of SM934 on IFN-γ-primed peritoneal macrophages *in vivo*, naïve female C57BL/6 mice were *i.p.* injected with 300 units of mIFN-γ according to the method described by Takeda et al [49], and simultaneously orally administrated with SM934 (10 mg/kg/day) for consecutive 3 days. Then, peritoneal exudative cells were collected.

### Biochemical parameters, serum anti-dsDNA antibody detection, renal histopathology and immunofluorescence

All examinations and scoring were carried out according to our previous descriptions [38]. Briefly, urinary protein concentrations were determined with Coomassie blue G dye binding assay. Urinary protein was scored according to the following criteria: 0/trace, <0.3 mg/ml; 1+, ≥0.3 mg/ml; 2+, ≥1 mg/ml; 3+, ≥3 mg/ml; 4+, ≥10 mg/ml; Blood urea nitrogen (BUN) concentration was determined by BUN test kit (Jianchen biotechnology, Nanjing, China) according to the manufacturer's instructions; For ELISA detection of serum anti-dsDNA IgG and isotypes, sera from aged female NZB/W F_1_ mice (10 months of age) was used as the standard. Titers of standard serum were defined as follows: total IgG, 1000 U/ml; IgG1, 170 U/ml; IgG2a, 340 U/ml; IgG3, 270 U/ml; Formalin-fixed kidney sections were embedded in paraffin and stained with hematoxylin and eosin (H&E), then were graded by two independent renal pathologists; Snap-frozen kidneys were used for immunofluorescence examination of IgG deposits.

### Flow cytometric analysis

Surface marker and intracellular transcription factor staining was conducted and analyzed according to our previous methods [38]. For intracellular cytokine staining, cells were incubated for 4 hours with phorbol 12-myristate 13-acetate (PMA, 50 ng/ml) and ionomycin (750 ng/ml) in the presence of Brefeldin A (BFA, 10 µg/ml). At the end of incubation, suspended cells were collected. Adherent cells were treated with 10 mM EDTA-PBS solution (pH = 7.2) for 5 minutes and then collected. Suspended and adherent cells were mixed together and blocked with FcγR blocker before extracellular staining for corresponding fluorescence-labeled surface antibodies. After surface staining, cells were fixed, permeabilized and stained for cytokines according to manufacturer's instruction (FoxP3 Staining Buffer set was used).

The following reagents were used: FITC- and PE-anti-mCD4 (GK1.5), FITC- and PE-anti-mCD3 (145-2C11), biotinylated- and FITC-anti-mTCRγδ (GL3), biotinylated-anti-mB220 (RA3-6B2), PE-anti-mCD8 (2.43), FITC- and PE-anti-mCD11c (HL-3), biotinylated- and PE-anti-mCD11b (M1/70), FITC-anti-mGr-1 (RB6-8C5), FITC-anti-mIFN-γ (XMG1.2), PE-anti-mIL-10 (JES5-16E3), PE-anti-mIL-17 (TC11-18H10), and PE-Rat IgG1 were all purchased from BD Pharmingen; FITC-anti-mF4/80 (BM8), PE-Cy5-anti-mouse/rat FoxP3 (FJK-16s), PE-Cy5- and PE-Rat IgG2a, PE-Rat IgG2b, and FoxP3 Staining Buffer Set were all purchased from eBioscience.

### Cell purification

#### Splenic CD4^+^ T cell

Purified polyclonal CD4^+^ T cells from splenocytes were enriched as described previously [38].

#### Splenic and peritoneal macrophage

To isolate splenic F4/80^+^ macrophages, splenocytes were blocked with saturated anti-m-CD16/32 (2.4G2, purified in-house), then reacted with FITC-anti-m-F4/80 mAb. After extensive washing, cells were incubated with anti-FITC microbeads (Miltenyi Biotec, Bergisch Gladbach, Germany) and positively selected. To acquire highly purified resulted cells, two rounds of positive selection were conducted. Purity of resulted cells was determined by flow cytometry analysis, and was consistently >98%.

To isolate peritoneal macrophages, naive female C57BL/6 mice were elicited by *i.p.* injection of 0.5 ml of 3% thioglycollate medium (TG, Difco, Detroit, MI). Three days later, adherent peritoneal exudative cells were obtained by peritoneal lavage using ice-cold phosphate-buffered saline, seeded in dishes, and collected by removing the non-adherent cells after 2 hours incubation at 37°C. Peritoneal macrophages from mIFN-γ-elicited C57BL/6 mice were enriched using similar protocol with thioglycollate-elicited ones.

### Cell stimulation and culture

Splenocytes from NZB/W F_1_ mice were cultured in 24-well flat-bottom plates coated with anti-CD3 mAb (145-2C11, 5 µg/ml) for 24 hours. Splenocytes of OVA-immunized mice were stimulated with OVA (100 µg/ml) for 24 hours. After incubation, supernatants were collected and stored at −20°C for ELISA assay.

Primary peritoneal macrophages were cultured in 48-well flat-bottom plates and stimulated without or with 50 unit/ml mIFN-γ in the absence or presence of SM934 for 24 hours. After incubation, supernatants were collected and stored at −20°C for ELISA assay.

Purified CD4^+^ T cells and F4/80^+^ macrophages were cultured together with the ratio of 4∶1 in 96-well flat-bottom plates in the presence or absence of OVA (100 µg/ml) for 72 hours. Cultures were pulsed with 0.5 µCi ^3^H thymidine per well for 8 hours before harvesting and assessed for ^3^H thymidine incorporation at 72 hours.

### Real-time PCR

Total RNA was isolated using Trizol reagent (Invitrogen, Carlsbad, CA, USA), reverse transcribed, and polymerase chain reaction amplified using specific primers. Real-time PCR was performed with SYBR Green PCR Reagents (Qiagen, Valencia, CA, USA) and a Continuous Fluorescence Detection System (MJ Research, USA), according to the manufacturer's instructions. Relative quantitation of mRNA expression was calculated as the fold increase in expression by using the ΔΔCt method. The forward and reverse primers for FoxP3 and HPRT were as follows: FoxP3 forward 5′-CCC ATC CCC AGG AGT CTT G-3′, FoxP3 reverse: 5′-ACC ATG ACT AGG GGC ACT GTA-3′; HPRT forward: 5′-GTT GGA TAC AGG CCA GAC TTT GTT G-3′, HPRT reverse: 5′-GAG GGT AGG CTG GCC TAT AGG CT-3′.

### ELISA for cytokines detection

Cytokines in sera or culture supernatants were assayed using the mouse IL-10, IL-12/23p40, TGF-β, IFN-γ, IL-2, IL-4, IL-17A ELISA kit (all from BD Pharmingen) according to the manufacturer's instructions.

### Western blot

Purified CD4^+^ T cells from mice of different groups were directly lysed in SDS sample buffer and boiled for 5 min at 100°C. Proteins were resolved by 10% SDS-polyacrylamide gel electrophoresis (PAGE), transferred to the nitrocellulose membrane (Amersham Pharmacia Biotech, Buckinghamshire, UK). The membranes were treated with 5% BSA-TBST buffer (TBS containing 0.1% Tween20) for 2 hours to block nonspecific binding, rinsed, and incubated with anti-BCL-2 (SC-492, Santa Cruz Biotechnology) or anti-GAPDH (Cell Signaling Technology). Signals were detected with HRP-conjugated anti-rabbit or anti-mouse IgG using the ECL system (Amersham Biosciences)

### Statistical analysis

Data were analyzed using Student's t-test and one-way analysis of variance (ANOVA) with Newman-Keuls multiple comparisons on post-tests. Nonparametric data (histological score) were analyzed using Mann-Whitney U test. Survival rate for treated group was compared with control group by log-rank test followed by Bonferroni adjustment. P<0.05 was considered to be statistically significant.

## Supporting Information

Table S1 Glomerular, tubular, vascular, interstitial damage, and glomerular IgG depositions were analyzed using a semiquantitative scoring system and scored by two pathologists.
(DOC)

Table S2 * *P*<0.05, ** *P*<0.01 versus vehicle.
(DOC)

Table S3 Glomerular, tubular, vascular, and interstitial damages were analyzed using a semiquantitative scoring system: −, no changes; ±, minimal changes; +, mild changes; ++, moderate changes; +++, marked changes.
(DOC)
