# Supplementary material for: SM934 Treated Lupus-Prone NZB×NZW F1 Mice by Enhancing Macrophage Interleukin-10 Production and Suppressing Pathogenic T Cell Development
Source: PLoS One. 2012 Feb 28;7(2):e32424. doi: 10.1371/journal.pone.0032424 (PMC3289663; doi:10.1371/journal.pone.0032424)
Supplement: Table S1 — Glomerular, tubular, vascular, interstitial damage, and glomerular IgG depositions were analyzed using a semiquantitative scoring system and scored by two pathologists. (DOC) [file pone.0032424.s001.doc]

**Supplementary Table 1**. Individual results of renal histology and IgG deposition of NZB/W F1 mice after 3 months of SM934 treatment.

|  | Histology (damage score) | | | | Glomerular IgG deposition |
| --- | --- | --- | --- | --- | --- |
| Mice | Glomerular | Tublular | Vascular | Interstitial |
| Vehicle 1# | +++ | + | + | + | +++ |
| Vehicle 2# | +++ | + | + | + | +++ |
| Vehicle 3# | +++ | + | ++ | + | +++ |
| Vehicle 4# | ++ | + | - | + | +++ |
| Vehicle 5# | + | - | - | + | ++ |
| Vehicle 6# | + | - | - | + | ++ |
| Vehicle 7# | ± | + | ± | + | +++ |
| Vehicle 8# | ++ | + | + | + | +++ |
| Vehicle 9# | ++ | ± | ++ | + | +++ |
| Vehicle 10# | + | - | - | - | + |
| Vehicle 11# | + | ± | ± | + | +++ |
| PNS 1#  PNS 2#  PNS 3#  PNS 4#  PNS 5#  PNS 6#  PNS 7#  PNS 8#  PNS 9#  PNS 10#  PNS 11#  SM934 10 mg/kg 1# | +  +  ±  +  ±  ±  -  ±  +  -  -  + | ±  -  -  +  -  -  -  -  ±  -  -  ± | -  ±  -  ±  +  -  +  ±  +  ±  -  - | +  +  ±  +  +  -  +  ±  +  -  ±  - | ++  +  +++  +++  ++  +  +++  ++  +++  +  +  ++ |
| SM934 10 mg/kg 2# | - | - | - | - | + |
| SM934 10 mg/kg 3# | + | - | ± | ± | +++ |
| SM934 10 mg/kg 4# | - | - | - | - | + |
| SM934 10 mg/kg 5# | ± | - | - | - | ++ |
| SM934 10 mg/kg 6# | - | - | - | + | ++ |
| SM934 10 mg/kg 7# | - | - | - | - | + |
| SM934 10 mg/kg 8# | - | - | - | - | ++ |
| SM934 10 mg/kg 9# | - | - | - | - | ++ |
| SM934 10mg/kg 10# | - | - | - | - | ++ |
| SM934 10mg/kg 11# | - | - | - | - | ++ |

Glomerular, tubular, vascular, interstitial damage, and glomerular IgG depositions were analyzed using a semiquantitative scoring system and scored by two pathologists.
