# Supplementary material for: SM934 Treated Lupus-Prone NZB×NZW F1 Mice by Enhancing Macrophage Interleukin-10 Production and Suppressing Pathogenic T Cell Development
Source: PLoS One. 2012 Feb 28;7(2):e32424. doi: 10.1371/journal.pone.0032424 (PMC3289663; doi:10.1371/journal.pone.0032424)
Supplement: Table S2 — * P<0.05, ** P<0.01 versus vehicle. (DOC) [file pone.0032424.s002.doc]

**Supplementary Table 2**. Proportion of splenocyte population

| Population | Vehicle | PNS | SM934,  10mg/kg |
| --- | --- | --- | --- |
| Total CD3^+^CD4^+^ (%) | 33.20 ± 0.44 | 34.15 ± 0.44 | 37.44 ± 2.94 |
| Total CD3^+^CD8^+^ (%) | 8.67 ± 2.63 | 13.43 ± 3.79 | 12.94 ± 1.40 |
| CD3^+^CD4^+^/ CD3^+^CD8^+^ | 3.62 ± 0.82 | 2.59 ± 0.67 | 2.90 ± 0.23 |
| CD3^-^B220^+^ (%) | 51.51 ± 1.48 | 43.4 ± 0.86* | 51.38 ± 0.33 |
| CD4^+^B220^+^ (%) | 3.05 ± 0.69 | 4.30 ± 0.50 | 6.38 ± 1.20* |
| CD4^+^FoxP3^+^ (%) | 7.49 ± 0.29 | 7.80 ± 0.43 | 9.15 ± 0.65* |
| CD44^low^CD62L^+^ in CD4^+^ (%) | 7.09 ± 2.23 | 8.41 ± 1.39 | 16.37 ± 2.66** |
| CD44^hi^CD62L^-^ in CD4^+^ (%) | 75.75 ± 7.1 | 69.75 ± 11.47 | 64.50 ± 4.2* |
| CD69^+^ in CD4^+^ (%) | 24.61 ± 4.08 | 24.75 ± 1.72 | 21.99 ± 2.64 |
| CD11b^+^Gr-1^hi^ (%) | 1.00 ± 0.31 | 1.39 ± 0.29 | 1.55 ± 0.37 |
| CD11b^+^Gr-1^low^ (%) | 6.48 ± 0.54 | 7.88 ± 2.12 | 7.34 ± 0.81 |
| CD11b^+^CD11c^+^ (%) | 5.25 ± 0.72 | 5.99 ± 1.92 | 5.43 ± 0.86 |

* *P* <0.05, ** *P* < 0.01 versus vehicle.
