# Supplementary material for: SM934 Treated Lupus-Prone NZB×NZW F1 Mice by Enhancing Macrophage Interleukin-10 Production and Suppressing Pathogenic T Cell Development
Source: PLoS One. 2012 Feb 28;7(2):e32424. doi: 10.1371/journal.pone.0032424 (PMC3289663; doi:10.1371/journal.pone.0032424)
Supplement: Table S3 — Glomerular, tubular, vascular, and interstitial damages were analyzed using a semiquantitative scoring system: −, no changes; ±, minimal changes; +, mild changes; ++, moderate changes; +++, marked changes. (DOC) [file pone.0032424.s003.doc]

**Supplementary Table 3**. Individual results of renal histology of NZB/W F_1_ mice after 6 months of SM934 treatment

|  | Histology (damage score) | | | | |
| --- | --- | --- | --- | --- | --- |
| Mice | Glomerular | Tubular | Vascular | Interstitial | |
| Vehicle 1# | +++ | ++ | +++ | | +++ |
| Vehicle 2# | +++ | +++ | +++ | | +++ |
| Vehicle 3# | ++ | - | + | | ++ |
| Vehicle 4# | ± | - | + | | + |
| Vehicle 5# | + | - | ± | | ++ |
| Vehicle 6# | ± | ± | ++ | | ++ |
| Vehicle 7# | +++ | +++ | ++ | | +++ |
| Vehicle 8# | + | + | ++ | | + |
| PNS 1# | ± | - | - | | ± |
| PNS 2# | ± | ± | - | | ± |
| PNS 3# | ± | - | - | | + |
| PNS 4# | - | - | ± | | ± |
| PNS 5# | + | - | - | | + |
| PNS 6# | - | + | +++ | | ++ |
| PNS 7# | + | - | + | | - |
| PNS 8# | - | + | ± | | - |
| PNS 9# | + | ± | ± | | + |
| PNS 10# | ± | - | - | | - |
| SM934 10mg/kg 1# | + | - | + | | ++ |
| SM934 10mg/kg 2# | + | ± | + | | ± |
| SM934 10mg/kg 3# | + | - | - | | ++ |
| SM934 10mg/kg 4# | - | - | - | | ++ |
| SM934 10mg/kg 5# | ++ | ++ | +++ | | +++ |
| SM934 10mg/kg 6# | ++ | ++ | + | | +++ |
| SM934 10mg/kg 7# | + | ± | - | | ++ |
| SM934 10mg/kg 8# | ++ | ++ | + | | ++ |
| SM934 10mg/kg 9# | - | - | - | | ± |
| SM934 10mg/kg 10# | ± | - | - | | - |
| SM934 10mg/kg 11# | - | - | - | | ± |

Glomerular, tubular, vascular, and interstitial damages were analyzed using a semiquantitative scoring system: -, no changes; ±, minimal changes; +, mild changes; ++, moderate changes; +++, marked changes.
